# Prolonged ST-Segment Elevation and Persistent Left Ventricular Dysfunction Assessed by Dual-Isotope Myocardial Scintigraphy in Takotsubo Syndrome: A Case Report

**DOI:** 10.7759/cureus.105130

**Published:** 2026-03-12

**Authors:** Satoshi Kurisu, Hitoshi Fujiwara

**Affiliations:** 1 Department of Cardiology, National Hospital Organization Hiroshima-Nishi Medical Center, Otake, JPN

**Keywords:** case report, echocardiography, electrocardiogram, japanese geriatrics, microvascular dysfunction, stress

## Abstract

Takotsubo syndrome is characterized by transient left ventricular (LV) systolic dysfunction in the absence of obstructive coronary artery disease. ST-segment elevation is frequently observed in the acute phase and typically resolves within several days, evolving into T-wave inversion. Prolonged ST-segment elevation is uncommon, and its clinical significance remains incompletely understood. Here, we report the case of an elderly woman with takotsubo syndrome likely triggered by a urinary tract infection. The initial electrocardiogram showed marked ST-segment elevation with terminal T-wave inversion in leads V_3_-V_6_, with a maximum elevation of 3 mm in lead V_4_. ST-segment elevation persisted for at least 20 days, accompanied by persistent severe LV wall motion abnormalities and systemic inflammation, as evidenced by sustained elevation of C-reactive protein. Coronary angiography revealed no obstructive coronary artery disease. Furthermore, dual-isotope myocardial scintigraphy using ^201^Tl and ^123^I-β-methyl-p-iodophenyl-pentadecanoic acid (BMIPP) revealed a marked perfusion-metabolism mismatch, with moderately reduced ^201^Tl uptake and severely reduced ^123^I-BMIPP uptake in the dysfunctional segments. This case suggests that recovery of LV function may occasionally be incomplete or substantially delayed, particularly in the context of systemic inflammation.

## Introduction

Takotsubo syndrome is characterized by transient left ventricular (LV) systolic dysfunction in the absence of obstructive coronary artery disease [1,2]. It can present with ST-segment elevation, which may mimic ST-elevation myocardial infarction and pose a diagnostic challenge [3]. The syndrome predominantly affects elderly women and is often triggered by physical or emotional stress. Although the condition is generally reversible, most patients show near-complete recovery of LV wall motion within a few weeks. Nevertheless, recent studies have suggested that LV systolic dysfunction in takotsubo syndrome may not always be reversible, with a subset of patients showing persistent LV systolic dysfunction, raising questions about the traditional concept of takotsubo syndrome [4-7]. In addition, the clinical significance of persistent electrocardiographic (ECG) abnormalities, particularly prolonged ST-segment elevation [8], remains poorly understood. Dual-isotope myocardial scintigraphy allows simultaneous assessment of myocardial perfusion and metabolism and may provide insight into myocardial stunning or metabolic impairment.

Here, we report the case of an elderly woman with takotsubo syndrome who exhibited prolonged ST-segment elevation, persistent LV dysfunction, and dual-isotope scintigraphic findings suggesting perfusion-metabolism mismatch [9,10].

## Case presentation

An 89-year-old woman with a history of dementia, renal calculi, chronic kidney disease, and a thyroid tumor under conservative management had been bedridden and residing in a long-term care facility. She was referred and admitted to our hospital after being diagnosed with a urinary tract infection based on urinalysis findings, following the development of fever and inability to take oral intake.

On admission, she was able to respond to verbal stimuli but appeared mildly lethargic (Glasgow Coma Scale score: E3V4M6). She did not report any chest symptoms. Her vital signs were as follows: blood pressure, 110/68 mmHg; heart rate, 88 beats per minute; respiratory rate, 22 breaths per minute; and oxygen saturation, 92% on room air. No jugular venous distension was noted. Laboratory tests showed leukocytosis and elevated C-reactive protein (7.59 mg/dL), along with renal dysfunction (blood urea nitrogen: 85 mg/dL, creatinine: 1.83 mg/dL). Creatine kinase was within the normal range, whereas troponin I was markedly elevated at 602 pg/mL. Thyroid function tests revealed a euthyroid state (Table 1).

**Table 1:** Laboratory data. NT-proBNP: N-terminal pro-B-type natriuretic peptide

Variable	Day 1	Day 20	Reference range
Blood count			
White blood cell count (/µL)	11.0 × 10^3^	6.5 × 10^3^	3.3–8.6 × 10^3^
Red blood cell count (/µL)	3.61 × 10^6^	3.64 × 10^6^	3.86–4.92 × 10^6^
Hemoglobin (g/dL)	10.1	10.7	11.6–14.8
Hematocrit (%)	31.6	33.5	35.1–44.4
Platelet count (/µL)	216 × 10^3^	148 × 10^3^	158–348 × 10^3^
Blood chemistry			
Total bilirubin (mg/dL)	0.29	0.41	0.4–1.5
Aspartate aminotransferase (U/L)	13	9	13–30
Alanine aminotransferase (U/L)	12	6	7–23
Lactate dehydrogenase (U/L)	219	110	124–222
Total protein (g/dL)	6.1	-	6.6–8.1
Albumin (g/dL)	2.4	2.3	4.1–5.1
Blood urea nitrogen (mg/dL)	85.0	18.8	8–20
Creatinine (mg/dL)	1.83	1.41	0.46–0.79
Sodium (mmol/L)	137	131	138–145
Potassium (mmol/L)	4.34	4.84	3.6–4.8
Chloride (mmol/L)	103	99	101–108
Creatine kinase (U/L)	81	10	41–153
Creatine kinase-MB (ng/mL)	6.1	14.7	<5
C-reactive protein (mg/dL)	7.59	7.52	<0.14
NT-proBNP (pg/mL)	50,744	-	<126
Troponin I (pg/mL)	602	-	<26.0
Thyroid-stimulating hormone (µIU/mL)	1.05	-	0.7–1.48
Free thyroxine (ng/dL)	1.05	-	0.61–4.23

**Figure 1:**
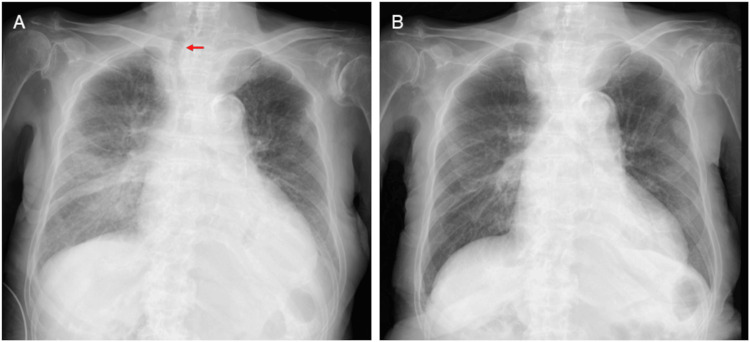
Serial chest radiographs. Initial chest radiography shows cardiomegaly and pulmonary congestion, together with rightward deviation of the trachea, presumably due to a thyroid tumor (A). Follow-up chest radiography on hospital day 20 demonstrates marked improvement in pulmonary congestion (B).

Chest radiography demonstrated cardiomegaly and pulmonary congestion, as well as rightward deviation of the trachea, presumably due to a thyroid tumor (Figure 1, Panel A, arrow).

**Figure 2:**
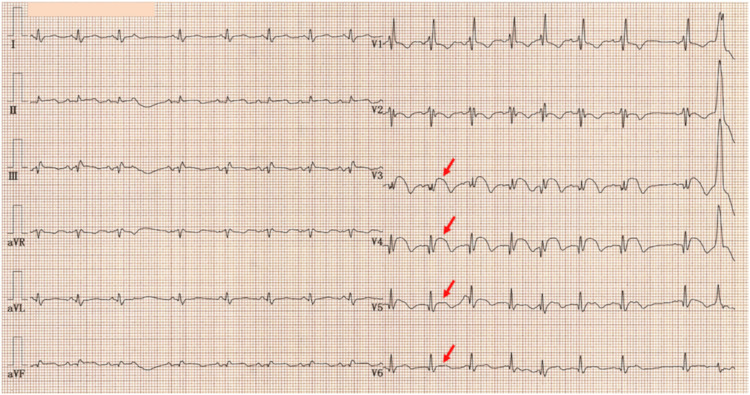
Initial electrocardiogram. Initial electrocardiogram showed right bundle branch block and marked ST-segment elevation with terminal T-wave inversion in leads V_3_–V_6_ (arrows), with a maximum ST-segment elevation of 3 mm in lead V_4_. Subtle ST-segment elevation less than 1 mm was also noted in the limb leads (I, II, III, -aVR, aVF), which was considered significant relative to the QRS amplitude. The corrected QT interval was prolonged at 494 ms.

ECG showed right bundle branch block and marked ST-segment elevation with terminal T-wave inversion in leads V_3_-V_6_ (Figure 2, arrows), with a maximum ST-segment elevation of 3 mm in lead V_4_. Subtle ST-segment elevation of less than 1 mm was also noted in the limb leads (I, II, III, -aVR, aVF), which was considered significant relative to the QRS amplitude. The corrected QT interval was prolonged at 494 ms.

**Figure 3:**
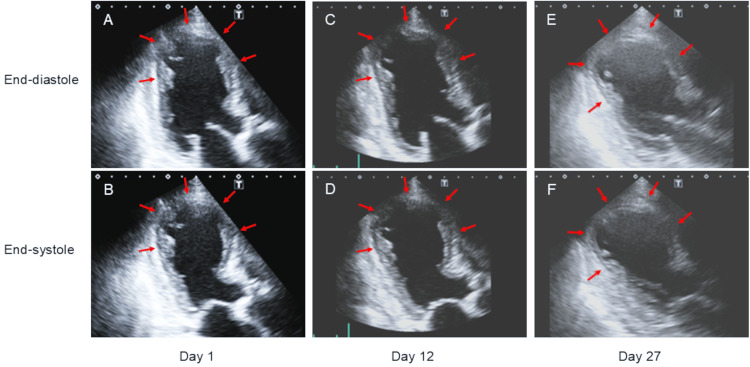
Serial echocardiographic images. Transthoracic echocardiography demonstrates extensive akinesis of the left ventricular (LV) apical segments, extending beyond the territory of a single coronary artery, with an ejection fraction of 35% (A and B, arrows). Follow-up echocardiography was performed weekly, demonstrating no significant recovery of LV wall motion throughout the hospital course (C-F, arrows). The LV ejection fraction remained between 35% and 40% during the hospital course, with no significant improvement.

Transthoracic echocardiography demonstrated extensive akinesis of the LV apical segments, extending beyond the territory of a single coronary artery, with an ejection fraction of 35% (Figure 3, Panels A and B, arrows).

**Figure 4:**
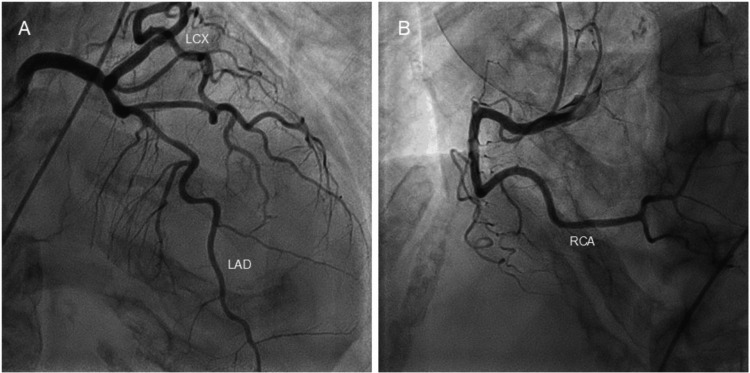
Coronary angiograms. Coronary angiography of the left (A) and right (B) coronary arteries was performed one week after admission, revealing no significant coronary artery stenosis. LAD: left anterior descending coronary artery; LCX: left circumflex coronary artery; RCA: right coronary artery

Based on the clinical course and the findings on ECG and echocardiography, takotsubo syndrome was considered the most likely diagnosis. The exact onset could not be determined, as the patient had no clearly identifiable chest symptoms. Transdermal bisoprolol was initiated to reduce myocardial oxygen consumption and prevent arrhythmias, together with intravenous antibiotic therapy for the urinary tract infection. Because the patient was unable to maintain adequate oral intake due to her general condition, she was managed with intravenous fluids and careful monitoring. After improvement of the patient’s volume status, coronary angiography of the left (Figure 4, Panel A) and right (Figure 4, Panel B) coronary arteries was performed one week after admission, revealing no significant coronary artery stenosis. These findings, together with the characteristic LV wall motion abnormalities, fulfilled the international takotsubo diagnostic criteria [1]. Although myocarditis could not be completely excluded because endomyocardial biopsy and cardiac magnetic resonance imaging were not performed, the overall clinical presentation was consistent with takotsubo syndrome.

**Figure 5:**
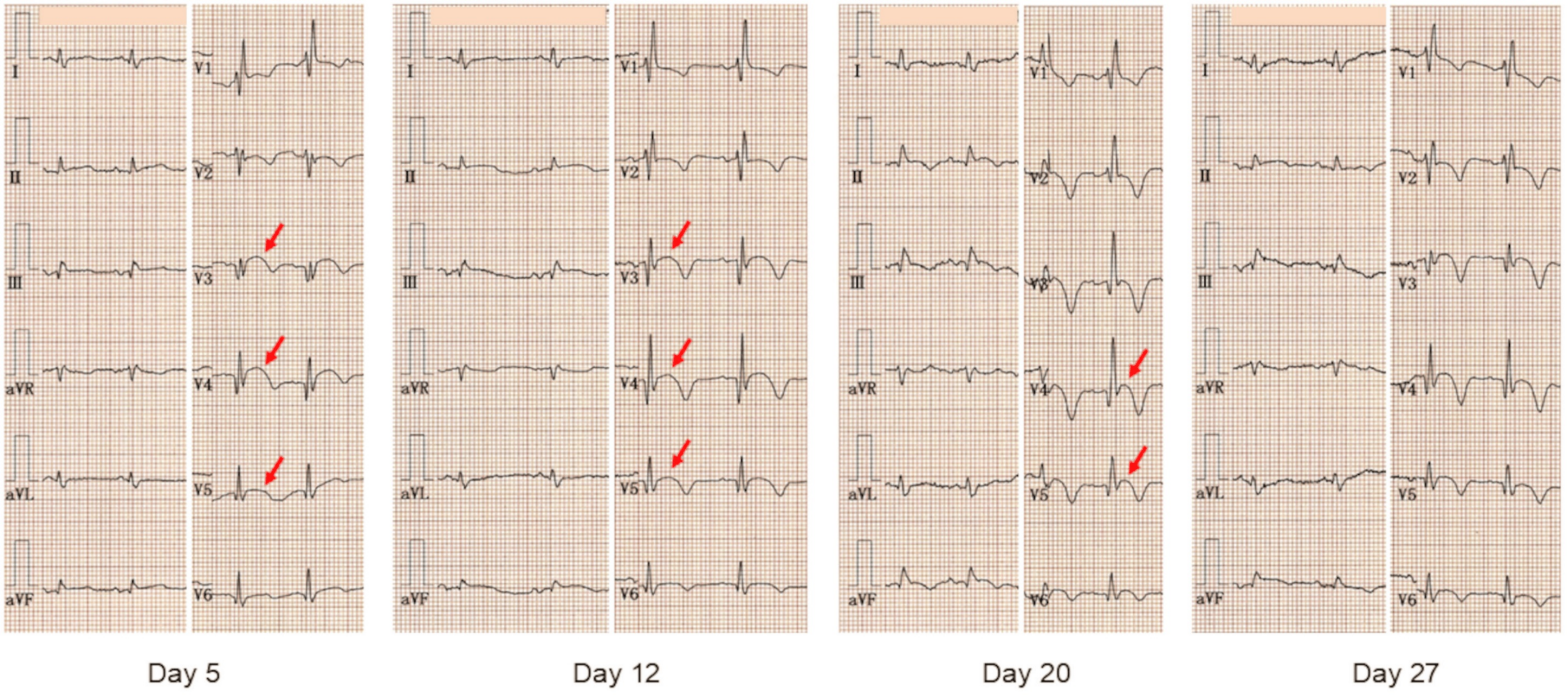
Serial electrocardiograms. Serial electrocardiograms demonstrated prolonged ST-segment elevation persisting through hospital day 20, which resolved by day 27 (arrows). T-wave inversion was also observed in both limb and precordial leads, with a maximum negative amplitude of 7 mm in leads V_3_ and V_4_.

Serial ECGs demonstrated prolonged ST-segment elevation persisting through hospital day 20, which resolved by day 27 (Figure 5, arrows). T-wave inversion was also observed in both limb and precordial leads, with a maximum negative amplitude of 7 mm in leads V_3_ and V_4_.

Throughout the clinical course, she did not report any apparent chest symptoms, and no significant arrhythmias were observed. Serial echocardiography performed weekly demonstrated no significant recovery of LV wall motion (Figure 3, Panels A-F). The LV ejection fraction remained between 35% and 40% during the hospital course, with no significant improvement.

**Figure 6:**
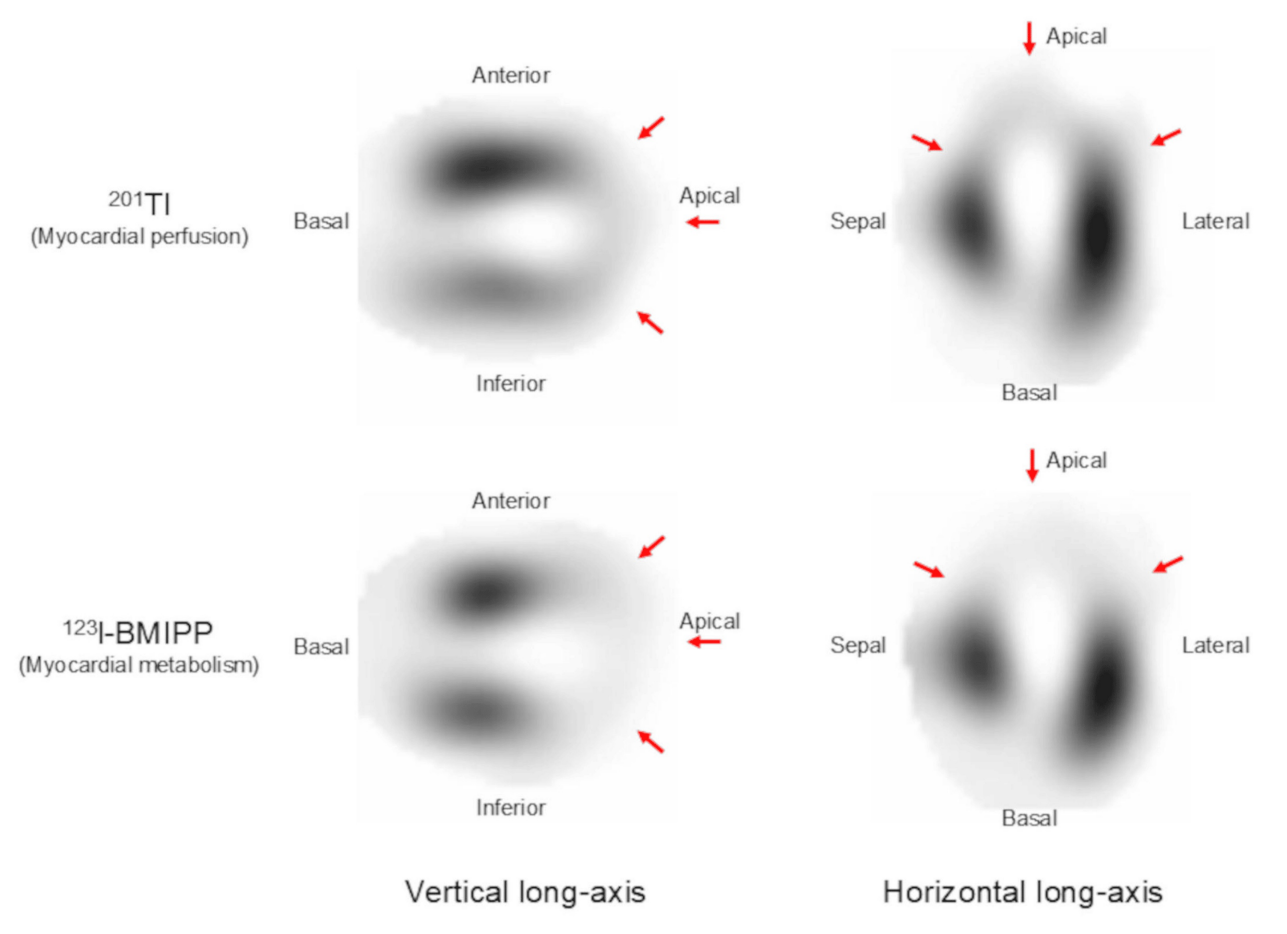
Dual-isotope myocardial scintigraphic images. Dual-isotope myocardial scintigraphy using ^201^Tl and ^123^I-β-methyl-p-iodophenyl-pentadecanoic acid (BMIPP) demonstrates a marked perfusion–metabolism mismatch, with moderately reduced ^201^Tl uptake and severely reduced ^123^I-BMIPP uptake in the dysfunctional segments.

On hospital day 20, the C-reactive protein level remained elevated at 7.52 mg/dL, whereas the creatine kinase level was within the normal range. Chest radiography showed marked improvement in pulmonary congestion (Figure 1, Panel B). Dual-isotope myocardial scintigraphy using ^201^Tl and ^123^I-β-methyl-p-iodophenyl-pentadecanoic acid (BMIPP) demonstrated a marked perfusion-metabolism mismatch, with moderately reduced ^201^Tl uptake and severely reduced ^123^I-BMIPP uptake in the dysfunctional segments (Figure 6).

She was discharged to the long-term care facility four weeks after admission with continued dependence on intravenous fluid therapy due to persistent inability to resume oral intake and died three months later from presumed natural causes related to advanced age.

## Discussion

In this report, we describe the case of an elderly woman with takotsubo syndrome who exhibited prolonged ST-segment elevation, persistent severe LV dysfunction, and characteristic dual-isotope scintigraphic abnormalities in the absence of obstructive coronary artery disease. In contrast to the typical clinical course of rapid recovery of LV function, our patient showed no significant improvement in LV wall motion even after one month.

Recent studies have highlighted the heterogeneity in the time course of functional recovery in takotsubo syndrome [4-7]. Matsushita et al. reported that a subset of patients exhibited incomplete recovery of LV function, which was associated with persistent systemic inflammation reflected by elevated C-reactive protein levels [5]. Similarly, Almendro-Delia et al. identified elevated C-reactive protein as an independent predictor of delayed recovery [7]. These findings suggest that systemic inflammation may contribute to delayed recovery of LV function in takotsubo syndrome [5-7]. In line with these observations, our patient showed sustained elevation of C-reactive protein during the acute and subacute phases, which may have contributed to persistent LV dysfunction. Despite growing evidence, the determinants and pathophysiological mechanisms underlying delayed or incomplete LV recovery in takotsubo syndrome, including ECG abnormalities, have not been fully elucidated [4-7]. ST-segment elevation is frequently observed in the acute phase of takotsubo syndrome and typically resolves within several days, evolving into T-wave inversion [1-3]. Prolonged ST-segment elevation lasting several weeks is uncommon. In the present case, ST-segment elevation persisted for at least 20 days. Kumar et al. reported that patients with cardiac rupture tended to be older and had a higher frequency of prolonged ST-segment elevation, suggesting that this finding may reflect more extensive myocardial involvement [8]. In the present case, although cardiac rupture was fortunately avoided, the prolonged ST-segment elevation may similarly reflect more extensive myocardial involvement.

Dual-isotope myocardial scintigraphy demonstrated severely reduced ^123^I-BMIPP uptake with relatively preserved ^201^Tl uptake in the dysfunctional segments, indicating a perfusion-metabolism mismatch. In this context, dual-isotope scintigraphy may provide additional insight into myocardial metabolic abnormalities. Previous studies have suggested that this mismatch pattern may represent transient myocardial metabolic impairment in the setting of coronary microvascular dysfunction in takotsubo syndrome [9,10]. Several invasive physiological studies have demonstrated increased index of microcirculatory resistance and impaired coronary flow reserve in the acute phase of takotsubo syndrome, highlighting the pivotal role of coronary microvascular dysfunction [11-13]. Among these, Warisawa et al. reported an elevated index of microcirculatory resistance in the acute phase, accompanied by a perfusion-metabolism mismatch, both of which normalized at the one-month follow-up, suggesting a close link between microvascular dysfunction and transient myocardial metabolic impairment [13]. Taken together, these findings suggest that sustained microvascular dysfunction and associated metabolic impairment may have contributed to the prolonged LV wall motion abnormalities observed in our patient.

This case has several limitations. Cardiac magnetic resonance imaging was not performed, precluding direct assessment of myocardial edema and fibrosis, and myocarditis could not be completely excluded, particularly in the context of infection and systemic inflammation. Serial troponin measurements were not available, which limited the evaluation of the temporal trend of myocardial injury markers. In addition, long-term ECG and echocardiographic follow-up were unavailable after discharge.

## Conclusions

We report a rare case of takotsubo syndrome in an elderly woman characterized by prolonged ST-segment elevation, persistent LV systolic dysfunction, and perfusion-metabolism mismatch on dual-isotope myocardial scintigraphy. Although takotsubo syndrome is generally considered a reversible condition, this case suggests that recovery of LV function may occasionally be incomplete or substantially delayed, particularly in the context of systemic inflammation. Therefore, careful ECG monitoring and follow-up cardiac imaging may be helpful in selected patients to assess recovery patterns and better understand the clinical course. Further studies are needed to clarify the mechanisms underlying such atypical presentations.
